# Long-term outcomes of tympanoplasty with persistent air-bone gap in adults with chronic otitis media: hearing, health care utilization and quality of life

**DOI:** 10.1007/s00405-025-09421-w

**Published:** 2025-05-06

**Authors:** Susan Arndt, Olivier Deguine, Jérôme Nevoux, Serafin Sánchez, Miray-Su Yılmaz Topçuoğlu, Christiane D’hondt, Håkan Hua, Ray Lin, Xabier Altuna

**Affiliations:** 1 Department of Otorhinolaryngology - Head and Neck Surgery, Faculty of Medicine, Medical Center - University of Freiburg, Killianstrasse 5, Freiburg, Germany; 2 Department of Otolaryngology, Purpan Hospital Toulouse, Place du Dr Baylac, Toulouse, France; 3 AP-HP, Department of Otolaryngology, Hôspital Bicêtre Paris Saclay, Le Kremlin- Bicêtre, France; 4 University Hospital Virgen Macarena, Avenida Dr. Fedriani 3, Seville, Spain; 5 Department of Otorhinolaryngology, University Hospital Heidelberg, Im Neuenheimer Feld 400, Heidelberg, Germany; 6 Cochlear Limited, 1 University Ave Macquarie University, Sydney, Australia; 7 Department of Otorhinolaryngology Head and Neck Surgery, University Hospital Donostia, San Sebastián, Spain

**Keywords:** Chronic otitis media, Hearing loss, Surgical outcomes, Quality of life, Aural rehabilitation

## Abstract

**Purpose:**

To evaluate long-term hearing outcomes, healthcare utilization, and health-related quality of life (HRQoL) in adults with chronic otitis media (COM) who underwent primary tympanoplasty (PT) but were left with a significant air-bone gap (25–30 dB).

**Methods:**

A retrospective medical chart review was conducted to assess the standard of care for COM patients across three European countries, analyzing hearing data, healthcare utilization, and demographics. Additionally, HRQoL and hearing disability were assessed prospectively using questionnaires. Sixty-nine adults diagnosed with COM who underwent PT between 2010 and 2016 were included.

**Results:**

Average hearing outcomes showed minimal to no improvement, with a substantial number of patients experiencing moderate to severe hearing loss post-PT. Many continued to rely on rehabilitative technologies, with bone conduction hearing device (BCHD) users reporting higher consistent use compared to conventional hearing aid users. Participants required an average of 7.6 healthcare visits with a mean follow-up time of 7.64 years after PT, underscoring the ongoing burden on healthcare systems. Impaired hearing negatively impacted both general and disease-specific HRQoL.

**Conclusion:**

Managing COM remains challenging due to the variability in surgical outcomes, particularly regarding hearing restoration. The high post-PT healthcare utilization and persistent HRQoL impairments caused by impaired hearing highlight the need for more effective rehabilitative strategies such as conventional hearing aids and BCHD.

Otitis media (OM) refers to a range of inflammatory conditions affecting the middle ear, including acute OM, which can progress to chronic otitis media (COM). COM is often complicated by the development of cholesteatomas—benign but potentially harmful growths in the middle ear caused by the accumulation of dead skin cells forming a cyst. Clinically, COM is characterized by recurrent middle ear and mastoid inflammation, often presenting as otorrhea due to tympanic membrane perforation, especially in chronic suppurative otitis media cases (CSOM). COM frequently originates from acute middle ear infections in childhood [1].

The World Health Organization defines OM as chronic if otorrhea persists for more than two weeks, whereas other guidelines define COM as ongoing tympanic membrane perforation with discharge lasting over six weeks to three months despite treatment [2]. Multiple factors contribute to the recurrence of COM, which represents a significant burden on global health, impacting patients’ quality of life (QoL) [3]. COM is one of the most prevalent chronic infectious diseases globally and a leading cause of conductive hearing loss (CHL) and mixed hearing loss (MHL) [4]. Global estimates suggest that COM affects between 65 and 330 million individuals, with approximately 60% experiencing hearing impairment [5]. The recurring infections may lead to erosion of the ossicles and ossicular discontinuity, a key feature of COM and a primary contributor to hearing loss, which can range from mild to severe (20–60 dB HL) and may be irreversible [6–8]. In children, this hearing loss can adversely affect speech development, learning, and behavior [9, 10], while in adults, it is linked to reduced QoL and higher rates of anxiety and depression [11]. If left untreated, COM can result in severe complications, such as brain abscesses and meningitis [12].

The multifactorial nature of COM poses treatment challenges, often involving bacterial and viral co-infections that complicate management [13]. The primary goals of treatment are to maintain a dry and infection-free ear and to restore hearing, typically through surgical interventions. Non-surgical treatments, such as earwax removal and topical or systemic antimicrobials, aim to control infection, but surgical options, including mastoidectomy and tympanic membrane reconstruction [14], are often necessary to remove diseased tissue and improve hearing [15]. In more complex cases, such as those involving cholesteatomas or ossicular discontinuity, invasive procedures like ossiculoplasty are required to restore the ossicular chain. The most commonly used prostheses in these procedures are partial ossicular replacement prostheses and total ossicular replacement prostheses. However, these surgeries carry risks, including potential permanent hearing damage [16], and there is significant variability in reported surgical outcomes [17]. Previous studies assessing prognostic factors of successful tympanoplasty have shown that both anatomic, surgical and technical elements diversely affect its functional outcome. For instance, factors such as the status of the mucosal lining, the performance of mastoidectomy, the availability of the malleus handle, and the size of the tympanic membrane perforation play a crucial role [18]. Additionally, surgeon experience, the use of an endoscope, cartilage grafting, and a longer pre-operative period without otorrhea have also been identified as significant predictors of surgical success [19].

To date, the methods for assessing hearing post-surgery are inconsistent, and there are no standardized protocols for evaluating hearing outcomes after COM surgery. Furthermore, the long-term clinical benefits of these interventions are poorly documented, with most publications emphasizing short-term surgical outcomes and hearing assessments. Although many surgeries result in a non-discharging ear, some patients do not experience satisfactory hearing restoration, and in certain cases, hearing may worsen postoperatively [20, 21]. The condition of the ossicular chain is a critical determinant of hearing outcomes. For instance, a retrospective study involving 213 patients found that those with an intact ossicular chain had significantly better long-term hearing outcomes after tympanoplasty [22]. Given the documented mild to moderate CHL in COM patients prior to surgery [23] and their risk of progressing to MHL [24], it is essential to thoroughly assess hearing outcomes in parallel to treatment of the infection. However, audiometric evaluations are not consistently performed across studies, leading to difficulties in comparing outcomes [25]. A recent review reported that successful tympanoplasty (types I-IV) defined as air-bone-gap (ABG) closure of ≤ 20 dB HL, restored hearing in only 70% of patients with COM-related hearing loss, leaving 30% without satisfactory rehabilitation [26]. This is a group that has received limited attention in the current literature.

In summary, while surgical treatment for COM primarily aims to achieve a safe, dry ear, the extent to which it results in long-term hearing improvement remains unclear. Furthermore, there is a lack of comprehensive data on the outcomes for patients who do not experience successful hearing improvement after primary tympanoplasty (PT). This study aims to fill this gap by characterizing long-term hearing outcomes and health-care utilization, and by assessing patient-reported quality of life in adults with a history of COM who have undergone PT and left with a large ABG (25–30 dB). Additionally, the study will explore socio-economic factors, demographics, and healthcare utilization patterns to provide a broader understanding of the impact of COM and its treatment.

## Materials and Methods

### Study population

A total of 92 subjects consented to participate in this clinical investigation. Of these, 69 subjects (75%) met the eligibility criteria and were included in the final analysis. The remaining 23 subjects were excluded due to deviations from the clinical investigation plan or non-compliance with Good Clinical Practice guidelines. Eligible participants were adults (18 years or older) diagnosed with COM who underwent PT between 2010 and 2016. In addition, inclusion criteria required hearing outcomes characterized by a pure tone average (PTA4) ABG of 30 dB or greater across four frequencies (0.5, 1, 2 and 4 kHz) or an ABG of 25 dB or greater accompanied by hearing loss exceeding 40 dB within 12 months post-PT.

Paediatric subjects were excluded from the study. The 69 eligible subjects were distributed across three countries and six different sites. In France, there were 17 participants with 11 participants from Hôpital Bicêtre (Paris) and 6 participants from Hôpital Purpan (Toulouse). In Germany, 20 participants were included. Of these, 14 participants were from Medical Center – University of Freiburg, and 6 participants were from University Hospital Heidelberg. In Spain, there were 32 participants. Hospital Universitario Donostia had the largest group, with 29 participants, while Hospital Universitario Virgen Macarena had 3 participants. The first subject’s informed consent was received on February 1, 2022, and the last subject’s visit was completed on October 9, 2023. Notably, none of the recruited participants had a history of cleft palate, Turner syndrome, or Down syndrome. Demographic and baseline characteristics for the 69 subjects are detailed in Table 1.

**Table 1:** Demographics of the study sample

Variable	*N* = 69
Age (years)	
Mean (SD)	50.8 (18.08)
**Gender n (%)**	
Female	37 (53.6%)
Male	32 (46.4%)
**Diabetes n (%)**	
No	65 (94.2%)
Yes	4 (5.8%)
**Cholesteatoma n (%)**	
No	33 (47.8%)
Yes	36 (52.2%)
**Duration of Follow-up (months)**	
Mean (SD)	91.7 (18.23)

## Measurements

### Hearing data

Hearing data was extracted from participants’ medical records, including information on etiology and hearing history. Hearing data was collected for three key timepoints: (1) pre-PT, (2) post-PT, and (3) the most recent available air conduction (AC) and bone conduction (BC) audiograms. PTA4 for AC and BC thresholds were calculated for unaided listening conditions, by averaging the hearing thresholds at 0.5, 1, 2, and 4 kHz. If the 4 kHz measurement was unavailable, the 3 kHz measurement was used as a substitute. When responses at all four frequencies were not available, the PTA4 was marked as missing. Where required, audiograms for certain participants that were collected at a partner hearing aid center rather than at the clinic, the clinic would contact the relevant audiologist to request a copy of the test results. Information of the usage of hearing device was also collected (e.g., conventional hearing aids or bone conduction implants).

### Health care service utilisation

To gather socio-economic data, demographics, and information on service utilization, the Adapted Client Service Receipt Inventory (CSRI) was employed. The CSRI is a widely used tool for collecting comprehensive data on the range of services and supports accessed by study participants. Developed in the mid-1980s, this research instrument focuses primarily on service utilization and has been applied in over 500 studies. One key feature of the CSRI is its flexibility, allowing data collection at multiple points within a study to track changes over time. Originally designed for in-person administration by trained interviewers to the service user or their carer, it has since been successfully adapted for postal self-completion by participants [27].

In the current study, the adapted CSRI data from the six months preceding study enrolment was collected. This included information on demographics (e.g., marital status, cohabitation, usual residence, education level), medical history (e.g., chronic diseases, smoking status, date of first diagnosis with chronic ear infection, current middle ear issues, recurrent ear infections), hearing rehabilitation (e.g., current hearing aids or devices, type of aid/device, and daily usage), healthcare utilization related to ear infections and hearing difficulties (e.g., number of face-to-face consultations, diagnostic tests, hospital admissions, medications), and employment status (e.g., employment status, work absence in days/hours). In addition to the CSRI, any COM-related interactions with healthcare providers for the operated ear, including the type and number of interventions or procedures, medications, therapies, examinations, as well as Middle Ear Risk Index (MERI) factors [28] and surgical outcomes were collected.

### 12-Item speech, spatial, and qualities of hearing scale (SSQ12)

The SSQ12 is a shorter (12-item) version of the original 49-item SSQ questionnaire that measures the self-reported auditory disability in everyday life. The SSQ12 was employed to measure disability in three key domains (speech, spatial and qualities of hearing), reflecting real-world hearing challenges. The scale ranges from 0 to 10, with higher scores indicating less difficulty in hearing. The SSQ12 has shown to provide similar results to the original version of SSQ [29].

### Health utilities index mark 3 (HUI-3)

General Health-Related Quality of Life (HRQoL) was assessed using the Health Utilities Index Mark 3 (HUI-3), a validated self-reported, multi-attribute health status system. The HUI-3 consists of 15 items that cover eight health dimensions: vision, hearing, speech, ambulation, dexterity, emotion, cognition, and pain/complaints [30]. It is highly sensitive to changes in HRQoL and is recommended for use in studies evaluating hearing interventions [31]. The structure of the HUI-3 allows for parametric testing, enabling comparisons across studies and datasets. Each participant’s responses generate a unique health state based on their selected levels for each dimension, which is represented as a sequence of numbers. Utility values range from 0.00 (a health state considered as death) to 1.00, representing full or perfect health. A change in the overall HRQoL score of 0.03 is deemed clinically significant, while changes of 0.03–0.05 in individual attribute scores are also considered clinically important [30].

### The chronic otitis media outcome test (COMOT-15)

The Chronic Otitis Media Outcome Test (COMOT-15) is a validated 15-item questionnaire designed to reliably assess disease specific HRQoL in patients with COM [32]. The instrument includes three subscales: ear symptoms (questions 1–6), hearing function (questions 7–9), and mental health (questions 10–13), which together form the overall score (questions 1–13). In addition, it includes one question evaluating the general impact of COM on HRQoL (question 14) and one question assessing the frequency of doctor visits due to COM in the past six months (question 15). The total score and subscale scores are transformed to a 0–100 scale by dividing the sum of raw scores by the total possible score range, then multiplying by 100. The lower the score, the better HRQoL.

### Procedure/design

This study was primarily designed as a retrospective, multicenter medical chart review to evaluate the standard of care for COM patients across three European countries: Germany, France, and Spain. The retrospective component involved medical reviews, including hearing data, healthcare utilization, and demographic information. Additionally, a smaller prospective component focused on patient-reported outcomes (PROs) collected via questionnaires and surveys. The current study was conducted in four stages. First, clinical personnel at each site screened eligible subjects. Second, informed consent was obtained from those who met the eligibility criteria. Third, participants completed the PROs, either at home or during a routine clinic visit if scheduled during the study period. Finally, clinical site personnel reviewed medical records at the respective clinics.

### Statistical analyses

The primary objective of the study was not to evaluate the differences between treatments nor the strengths of associations. It was only essential that the study could capture all potential interventions offered as standard care for the selected population. The goal was to enrol 200 participants (based on previous sample size calculations using established methods for retrospective studies). However, due to slower-than-anticipated recruitment, and the value of the data already collected, recruitment was concluded after enrolling 92 participants.

All collected data was summarized descriptively, with selected variables visualized using appropriate graphical methods. Missing data for HUI3, SSQ12, and COMOT-15 were imputed according to the guidelines provided in their respective operational manuals. PTA4 was calculated by averaging hearing thresholds at 0.5, 1, 2, and 4 kHz. If the 4 kHz measurement was unavailable, the 3 kHz measurement was used as a substitute. For time to event related endpoints, if the day part of a date is missing, it will be set to the 15th. If the day and month part of the date is missing, it will be set to the 1st of July. There was no missing data imputation other than what is described above.

### Ethical considerations

This investigation adhered to the ethical principles outlined in the Declaration of Helsinki and the ISO 14155:2020 standard [33]. Prior to any study-related procedures, all subjects provided informed consent after being given ample time to consider participation and having their questions fully addressed. The study was registered on Clinicaltrials.gov with identifier NCT04864912.

## Results

### Hearing outcomes

#### Air-bone-gap closure after primary tympanoplasty

**Fig. 1:**
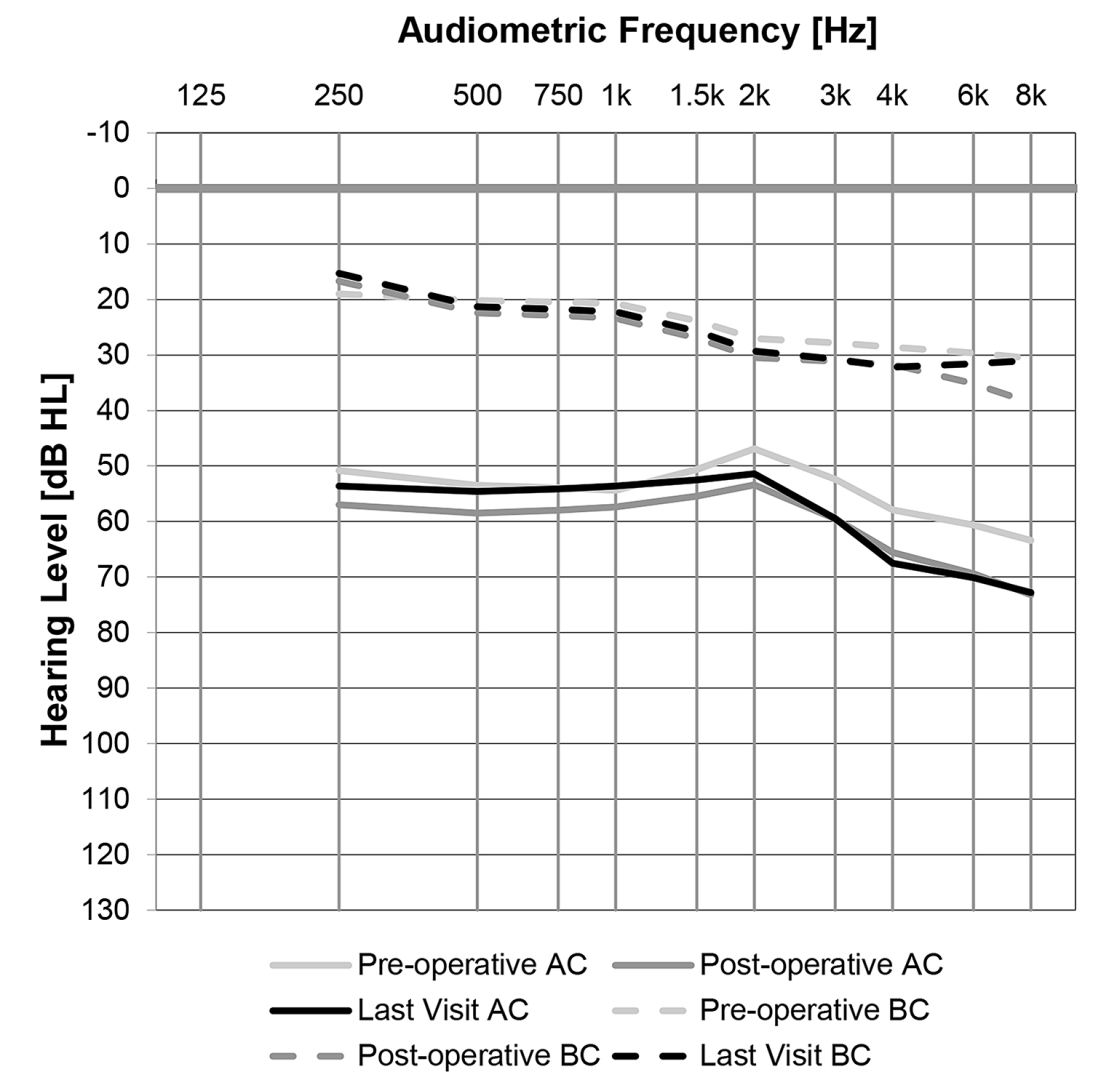
Air (solid lines) and bone (dashed lines) conduction hearing thresholds pre-tympanoplasty (light grey), post-tympanoplasty (dark grey) and at last visit (black)

Figure 1 illustrates mean hearing thresholds measured before and after PT, as well as at the final follow-up visit, which occurred on average 7.64 years after PT.

The mean pre-operative AC PTA4, as shown in Fig. 1, was 53.2 dB HL (SD: 19.64, range: 16.2–116.2), while the mean post-operative AC PTA4 was 58.7 dB HL (SD: 14.90, range: 37.5–102.5). This reflects a minor change in AC PTA4 of 5.5 dB (SD: 11.25, range: -23.8 to 41.2) following a PT surgery. These results were consistent with the mean AC PTA4 of the most recent audiogram, which was 56.7 dB HL (SD: 21.16, range: 16.0–120.0), showing an average change of 4.0 dB (SD: 15.50, range: -41.2 to 41.8) compared to the pre-operative values. Overall, these findings indicate non-clinically significant changes in hearing before and after surgery, and when compared to the latest follow-up for patients who underwent PT.

Similarly, for BC thresholds, the mean pre-operative BC PTA4 was 23.8 dB HL (SD: 14.76), and the mean post-operative BC PTA4 was 25.3 dB HL (SD: 14.85, range: 5.0–77.5), indicating a minor change of 0.6 dB (SD: 5.86, range: -13.2 to 12.8). This analysis is based on 37 subjects with available pre- and post-operative BC thresholds. The mean BC PTA4 from the most recent audiogram was 25.2 dB HL (SD: 13.89, range: 3.5–62.5), with an average change of 3.9 dB (SD: 8.77, range: -12.8 to 22.5, *n* = 31) compared to the pre-operative BC PTA4.

**Fig. 2:**
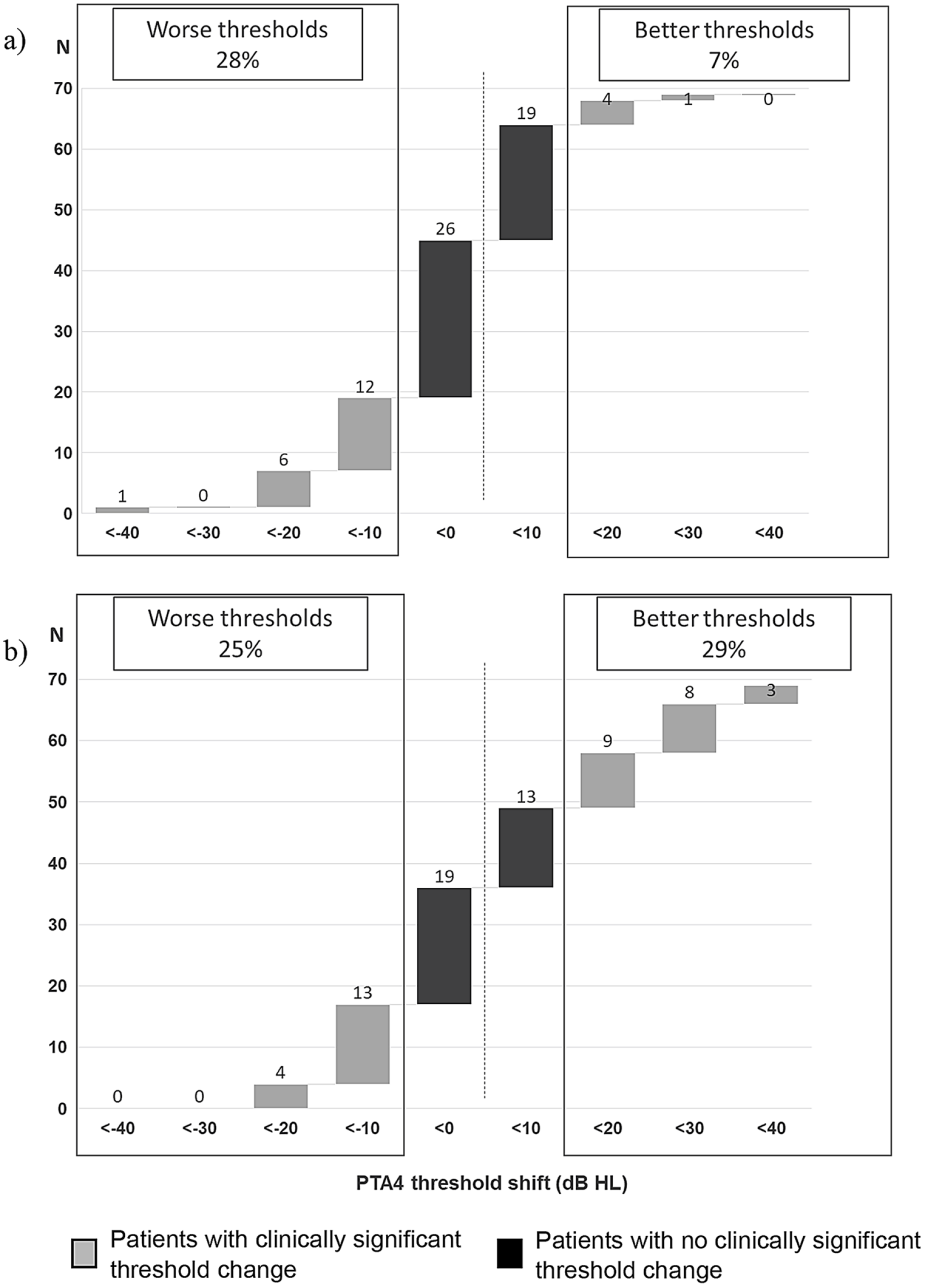
Change in air conduction thresholds (**a**) pre-operative primary tympanoplasty vs. post-operative primary tympanoplasty and (**b**) pre-operative primary tympanoplasty vs. latest audiogram

An analysis of changes in AC hearing levels before and after primary PT revealed that 65% of participants experienced no clinically significant shift in hearing thresholds (± 10 dB HL). Meanwhile, 28% showed worsened hearing thresholds (> 10 dB HL), and only 7% demonstrated an improvement (< 10 dB HL). A comparison between pre-operative PT and the latest audiogram showed slightly better outcomes, with 29% reporting improved hearing, 46% maintaining stable thresholds (± 10 dB HL), and 25% experiencing a decline. Overall, most patients with COM undergoing PT exhibit either no change or a deterioration in hearing thresholds, both post-operatively and when compared to latest audiogram (Fig. 2). Lastly, when controlling for AC hearing levels, four cases obtained normal hearing levels. However, 80% of patients still had a moderate to severe hearing loss 6–12 years after PT as indicated by the hearing levels in Fig. 1.

#### Hearing rehabilitation

At the end of the follow-up period, 57% (*n* = 39) of participants used some form of amplification, with 48% (*n* = 33) using hearing aids and 9% (*n* = 6) using bone conduction implants. Among those fitted with hearing aids, 70% reported regular use, while all participants with bone conduction implants (100%) reported consistent use. The six subjects received seven bone conduction implants following their PT, either in the operated or opposite ear. Only three subjects received bone conduction systems in the PT ear—one Baha Attract (with an unknown sound processor), one BONEBRIDGE (with a Samba Sound Processor), and one Ponto (with a Ponto 3 Superpower Sound Processor)—accounting for 4.3% of the total eligible study population. On average, participants indicated a high daily usage of their hearing devices, averaging 13.2 h per day.

### Health care service utilisation

For surgical procedures, participants had an average of 1.6 healthcare contacts (SD: 1.0, range: 1–5) during the follow-up period. Of the 69 participants, 45 (65.2%) underwent one surgical procedure, 14 (20.3%) had two, 5 (7.2%) had three, 3 (4.3%) had four, and 2 (2.9%) underwent more than four surgeries. These numbers include the PT, meaning participants who had only one procedure underwent a PT. For audiological assessment, 4.2 (SD: 4.75, range: 2–23) contacts on average were related to the ear affected by the PT. Of these contacts, 36.2% were with an audiologist, while 65.2% were with a physician. Among the 69 participants, 45 (65.2%) had two audiological assessments, 5 (7.2%) had three, 3 (4.3%) had four, and 16 (23.2%) had more than four assessments. Notably, the results obtained showed that all participants had at least two contacts for audiological assessment.

On average, participants had 7.6 other healthcare contacts (SD: 5.29, range: 1–22) related to the ear that underwent the PT. Of the 53 participants with additional healthcare contacts, 5 (9.4%) had one, 1 (1.9%) had two, 4 (7.5%) had three, 7 (13.2%) had four, and 36 (67.9%) had more than four contacts. The majority (73.9%) of these contacts were for counselling visits. Other reasons for healthcare contacts included hearing aid fitting and counselling (7.2%), ear pain (1.4%), study enrolment (1.4%), and hearing aid verification (1.4%). In sum, the current findings indicate that most subjects underwent only one surgical procedure, specifically PT, and had regular healthcare follow-ups, particularly for audiological assessments and counselling.

### Patient reported outcomes

#### Health utilities index mark 3 (HUI-3)

**Fig. 3:**
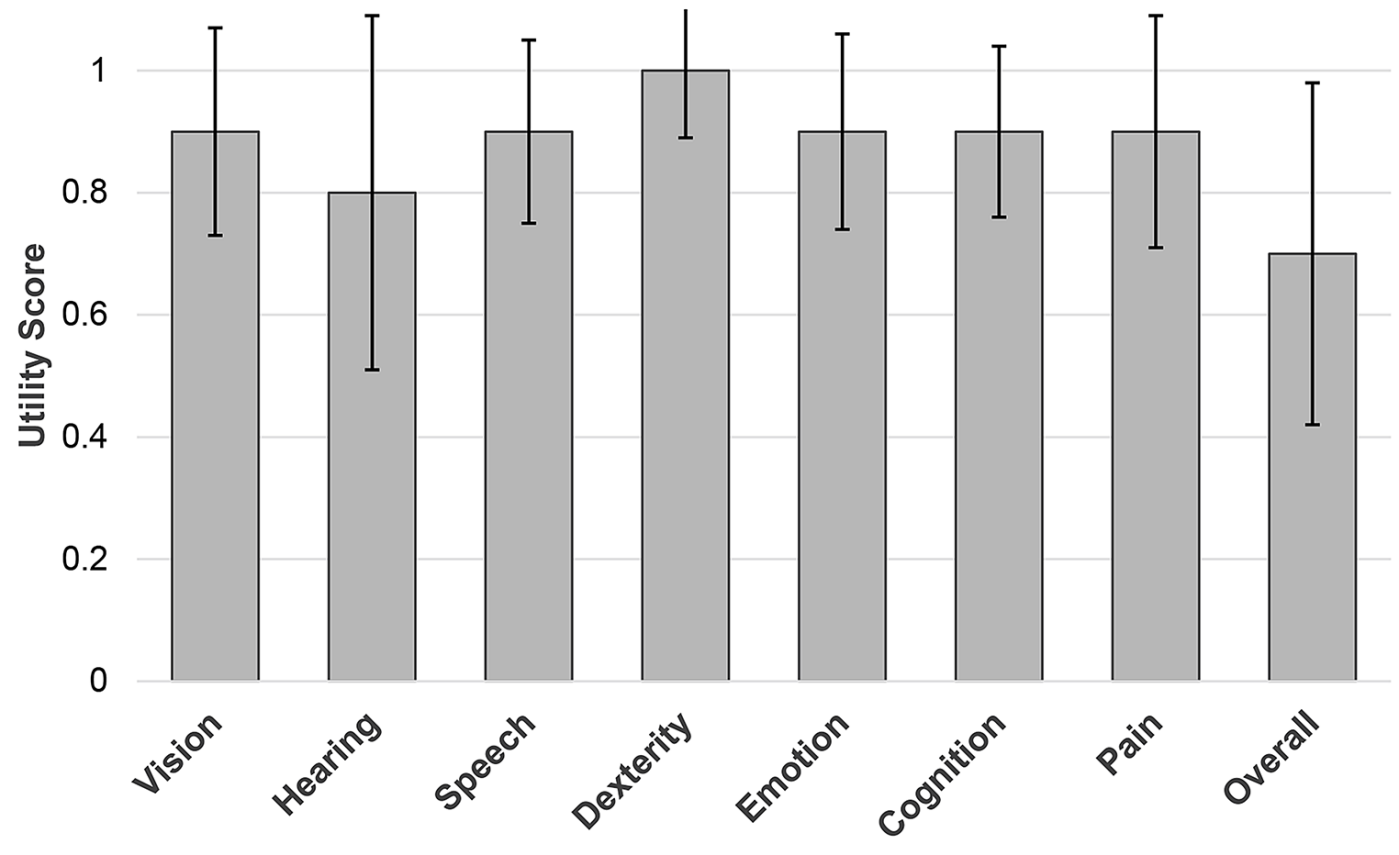
Overall mean score of the Health Utilities Index Mark 3

Figure 3 presents the overall mean score of the HUI-3 and its corresponding attributes. Higher scores indicate better HRQoL. The overall HUI score was 0.7 (SD: 0.28, range: 0–1). The mean scores for the individual attributes were as follows: Vision 0.9 (SD: 0.17, range: 0.0–1.0), Hearing 0.8 (SD: 0.29, range: 0.0–1.0), Speech 0.9 (SD: 0.15, range: 0.4–1.0), Dexterity 1.0 (SD: 0.11, range: 0.2–1.0), Emotion 0.9 (SD: 0.16, range: 0.0–1.0), Cognition 0.9 (SD: 0.14, range: 0.3–1.0), and Pain 0.9 (SD: 0.19, range: 0.0–1.0). No significant differences emerged between any of the subscales. While the findings suggest a somewhat restricted HRQoL for the study sample, it is noteworthy that the Hearing attribute had the lowest average score of 0.8.

#### Speech, spatial and qualities of hearing scale (SSQ)

**Fig. 4:**
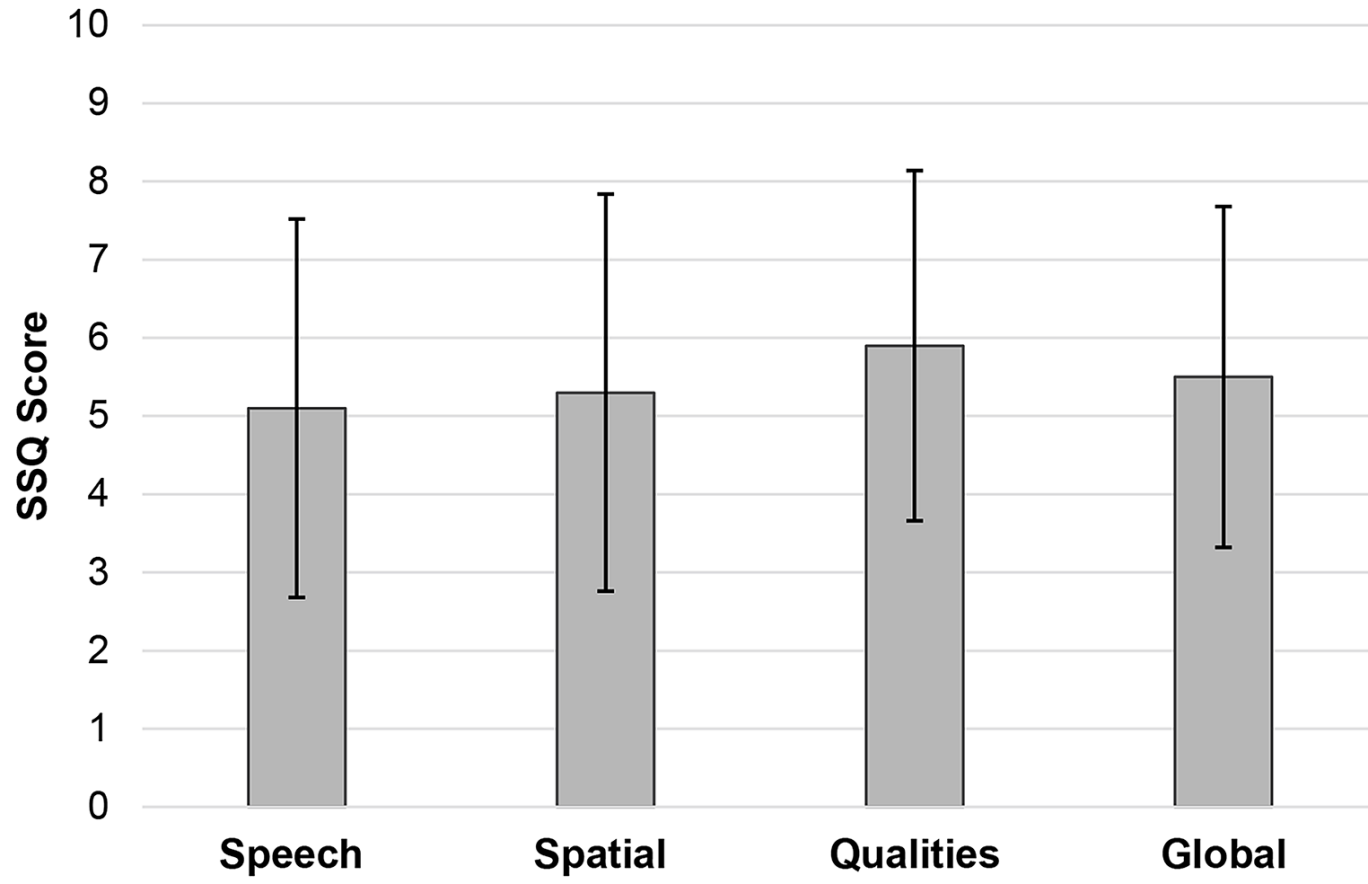
Overall mean score of the Speech, Spatial and Qualities of hearing scale

Figure 4 highlights overall mean score of the SSQ. Higher scores indicate better hearing functionality. The global SSQ12 score averaged 5.5 (SD: 2.18, range: 1.2–9.4). The sub-scores for speech, spatial, and sound quality were 5.3 (SD: 2.54, range: 0.0–9.8), 5.1 (SD: 2.42, range: 0.0–9.0), and 5.9 (SD: 2.24, range: 1.0–10.0), respectively. Thus, the current findings show that adults with COM report moderate disability in the speech, spatial and quality listening in everyday life.

#### The chronic otitis media outcome test (COMOT-15)

**Fig. 5:**
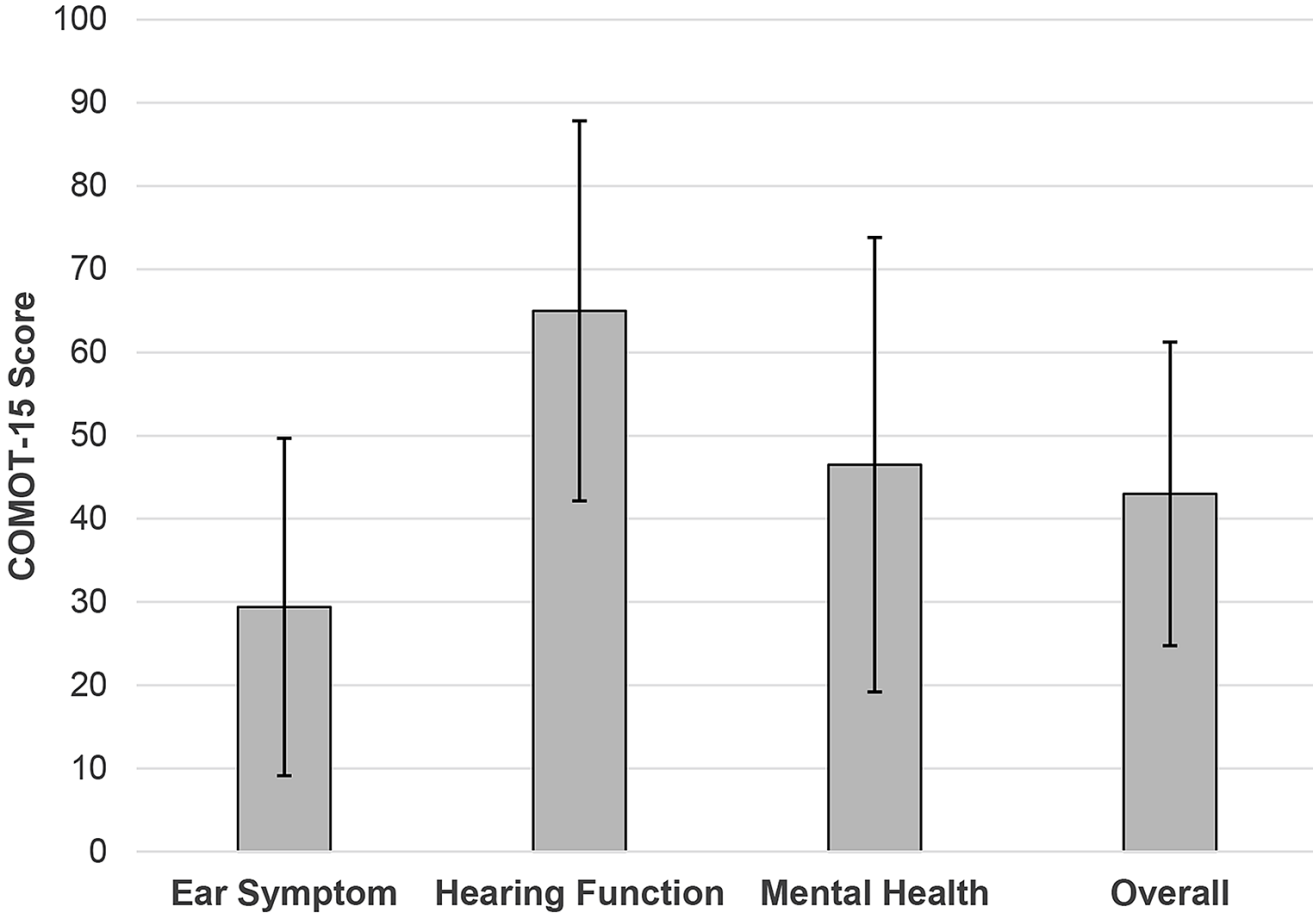
Overall mean score of the Chronic Otitis Media Outcome Test

Figure 5 shows the mean score of COMOT-15. A total of 68 subjects completed the questionnaire. Lower scores indicate better disease specific HRQoL. The mean overall score was 43.0 (SD = 18.25), with scores ranging from 3.1 to 78.5. The mean scores for the three subscales were as follows: ear symptoms, 29.4 (SD = 20.28, range: 0.0 to 96.7); hearing function, 65.0 (SD = 22.84, range: 6.7 to 100.0); and mental health, 46.5 (SD = 27.31, range: 0.0 to 100.0). Thus, consistent with post-operative hearing data, health care utilization, and the results from the HUI-3 and SSQ, disease-specific assessments of HRQoL by the COMOT-15 indicate that hearing ability is significantly impaired in patients with COM.

## Discussion

This study explored long-term hearing outcomes, healthcare utilization, and PROs in adults who underwent PT for COM. The findings highlight the complexity of managing COM and the variability in surgical outcomes, particularly regarding hearing restoration. While PT aims to repair structural damage caused by COM and to create a safe and dry ear, a significant portion of patients experienced either no improvement or a deterioration in hearing thresholds over time. The current study focused on patients with a substantial ABG after PT, with 52.2% of participants also diagnosed with cholesteatoma. This aligns with existing literature showing that cholesteatoma frequently complicates COM cases [34].

One notable outcome was the minimal change in AC and BC hearing thresholds post-PT. On average, patients exhibited a 5.5 dB worsening in AC hearing thresholds after surgery, with a modest 4 dB improvement at the latest follow-up compared to pre-surgery levels—an outcome not clinically significant. In particular, over 65% of participants showed no clinically meaningful improvement in hearing, underscoring the difficulty of restoring auditory function in COM patients, especially those with a large ABG after PT. Additionally, 28% of patients experienced further hearing deterioration, raising concerns about the long-term efficacy of PT as a treatment for COM. The average post-operative ABG PTA4 was 35 dB—6.3 dB worse than the pre-operative measurement—indicating that PT was generally unsuccessful in closing the ABG or improving hearing.

These findings align with previous research, such as Lewis et al. [26], which noted 30% of COM patients did not achieve an ABG closure of < 20 dB. The persistence of moderate to severe hearing loss in over 80% of patients further suggests that PT alone may not adequately address the auditory deficits associated with COM, particularly in cases with ossicular damage or cholesteatoma involvement. However, it is important to note that the primary goal of tympanoplasty is to create a safe, infection-free ear, with hearing restoration being a secondary aim. The focus of patients with a large ABG most likely influenced the observed outcomes. Despite disappointing hearing results, we believe achieving infection control remains crucial. Future research should explore factors contributing to poor hearing outcomes, such as patient age, disease severity, and surgical technique, to refine treatment strategies and improve long-term results.

The study also revealed substantial healthcare utilization among COM patients following PT. On average, participants underwent 1.6 surgical procedures, with some (7%) requiring up to four additional operations or more. Frequent follow-up visits were common, often involving multiple audiological assessments and counselling sessions. According to the CSRI, numerous ear-related healthcare visits to general practitioners, local ENT specialists, and audiologists were reported in the past six months. This elevated level of healthcare engagement underscores the ongoing burden COM imposes on both patients and healthcare systems. Even with surgical intervention, significant post-operative care is often necessary, including audiological evaluations, hearing aid fittings, and counselling, highlighting the complexity of managing this condition.

Among the participants, 57% relied on hearing devices after post-PT to manage residual hearing loss, while the remaining 43% either did not require hearing devices or did not benefit from them, depending on the condition of their contralateral ear. Despite these interventions, many participants continued to face significant hearing challenges, as evidenced by their SSQ12 scores and frequent healthcare utilization. Surprisingly, only three participants received bone conduction implants (in the PT ear), despite the high incidence of chronic ear infections. Bone Conduction Hearing Devices (BCHDs) effectively bypass the external and middle ear, providing a viable rehabilitation option for patients with conductive or mixed hearing loss [35]. Interestingly, BCHD users reported more consistent usage than those with conventional hearing aids, suggesting that bone conduction therapy may be a more effective alternative for certain patients. Additionally, bone conduction therapy may provide a higher user-comfort for patients with COM, as BCHD therapy allows the ear canal to be left open. Findings from de Wolfe et al. [36] suggest that for patients with MHL, especially those with a large ABG around 30–35 dB, speech recognition outcomes are often better with BCHDs compared to conventional hearing aids. This aligns with the current study’s observation that BCHD users reported more consistent usage and potentially better hearing outcomes, further emphasizing the need to explore BCHDs as a standard rehabilitative option for COM patients with significant hearing loss after PT.

The findings on PROs present a nuanced view. While generic HRQoL, measured by the HUI-3, was somewhat restricted, hearing-specific QoL stood out as the lowest subscale (mean score: 0.8). The study population exhibited moderate to severe hearing loss with a mild sloping sensorineural component, a level known to impair QoL [37, 38]. Notably, 43% of participants did not use any hearing device, raising additional concerns. As highlighted by Arlinger [39], untreated hearing loss can lead to reduced social activity, feelings of isolation, and an increased risk of depression. The SSQ12 results further corroborated these findings, indicating moderate hearing disability, suggesting ongoing challenges in daily life even after surgical intervention and rehabilitation. Comparatively, in a study assessing SSQ12 of users (*N* = 51) with active transcutaneous bone conduction hearing system, of which approximately 50% had a chronic ear infection, the results showed a mean change of SSQ scores between 2.0 and 3.0 points pre- vs. post-implantation [40]. One could thus speculate that if a majority of the current study sample had used a BCHD, lower (i.e. better) scores in the SSQ might have been obtained.

The COMOT-15 was used to assess disease-specific symptoms, with hearing function emerging as the primary subscale where subjects reported the most significant negative impact on HRQoL. This finding aligns with both the audiological data and PROs observed in this study. In a previous study by Baumann et al. [41], the COMOT-15 was compared to the Short Form-36 (SF-36) questionnaire, which, like the HUI-3, measures general HRQoL. The authors found that tympanoplasty significantly improved disease specific HRQoL in patients with CSOM, while general HRQoL remained unchanged. Nevertheless, it is important to note that, unlike the HUI-3, the SF-36 does not specifically measure hearing-related QoL. Therefore, future research should consider incorporating the COMOT-15 and/or HUI-3 to capture a more comprehensive view of patient experiences. This approach would provide a nuanced understanding of PROs and help tailor post-surgical care to better address the specific needs of patients with COM.

Lastly, limitations must be considered. The retrospective design limits the ability to establish causality or identify factors contributing to poor hearing outcomes post-PT. While the descriptive analysis provides valuable insights, future research should adopt prospective designs with larger, more diverse samples to explore these relationships in greater detail. The relatively small sample size also limits generalizability, although the multicentre design does offer a broad perspective on COM management across different healthcare systems. This study specifically targets a unique patient population: individuals with a significant ABG following PT. Our primary interest was to assess the long-term outcomes in this group. Remarkably, we found that even after extended follow-up periods—spanning up to 12 years—the majority of these patients maintained the same level of hearing achieved after their initial tympanoplasty, underscoring the important contribution of the obtained reference data specific to this patient group. Nevertheless, it should be noted that approximately 90% of the broader patient group appear to be doing well, with some even managing without the need for hearing devices. In the current study, we observed comparable outcomes between subjects with and without cholesteatoma across all outcome measures. However, the analysis did not address prognostic factors that may hinder the achievement of optimal functional outcomes following PT, nor was that the purpose of this study. Future investigations should explore these variables—including patient-specific anatomical variations and technical limitations—to enhance surgical strategies and improve prognostic accuracy. Moreover, studies that further examine intergroup differences will be valuable in tailoring individualized treatment approaches and optimizing management strategies for patients with varying underlying conditions and clinical profiles.

In conclusion, hearing outcomes, as measured by both audiometric data and PROs, indicate that patients with a failed tympanoplasty, indicated by ABG of 30 dB or larger, experience minimal to no improvement in hearing long-term, even after additional surgeries, with many continuing to rely on hearing aids and other rehabilitative technologies. Healthcare utilization remains high post-PT, highlighting the ongoing burden on both patients and healthcare systems. The prevalent use of conventional hearing aids, despite their potential to exacerbate infections due to the closed-ear design, and the limited adoption of BCHDs indicate a critical need for more effective rehabilitative options to address residual hearing loss. This study underscores the importance of robust clinical evidence to guide surgeons in determining the optimal timing for bone-conduction device implantation, which may offer more stable, long-term hearing outcomes for patients with failed tympanoplasty.

## Data Availability

Aggregated, anonymized study data will be made available upon reasonable request by contacting the corresponding author.
